# Kinetics of Secoisolariciresinol Glucosyltransferase LuUGT74S1 and Its Mutants

**DOI:** 10.1021/acs.jafc.4c06229

**Published:** 2024-08-30

**Authors:** Sadiq Saleh Moree, Lukas Böhm, Thomas Hoffmann, Wilfried G. Schwab

**Affiliations:** † Biotechnology of Natural Products, Technische Universität München, Liesel-Beckmann-Str. 1, Freising 85354, Germany; ‡ Department of Biochemistry, University of Thamar, P.O. Box 87246, Sana’a-Tiaz Road, Thamar 87246, Yemen

**Keywords:** flax, lignan, LuUGT74S1, mutants, SECO (secoisolariciresinol), SDG (secoisolariciresinol diglucoside), SMG (secoisolariciresinol monoglucoside)

## Abstract

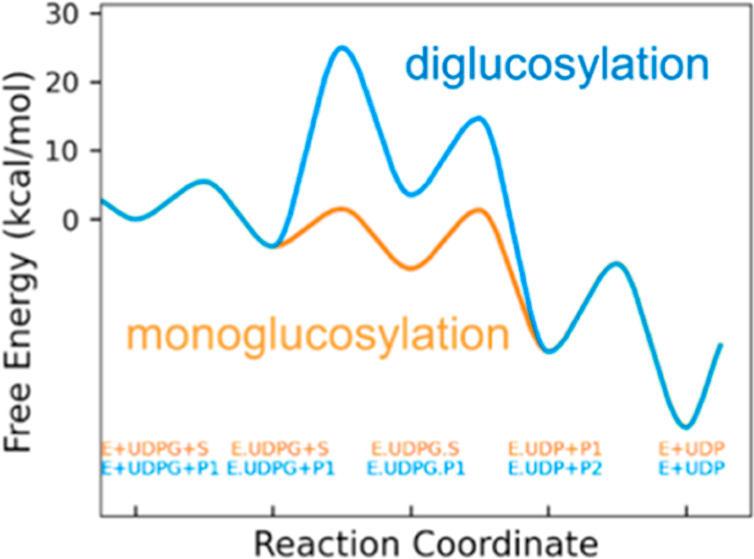

The lignan secoisolariciresinol (SECO) diglucoside (SDG) is a phytoestrogen with diverse effects. LuUGT74S1 glucosylates SECO to SDG, whereby only small amounts of the monoglucoside SMG are formed intermediately, which exhibit increased activity. To identify critical amino acids that are important for enzymatic activity and the SMG/SDG ratio, 3D structural modeling and docking, as well as site-directed mutation studies, were performed. Enzyme assays with ten mutants revealed that four of them had identical kinetic data to LuUGT74S1, while three showed reduced and one increased catalytic efficiency *k*_cat_/*K*_m_. S82F and E189L substitutions resulted in the complete absence of activity. A17 and Q136 are crucial for the conversion of SMG to SDG as A17S and Q136F mutants exhibited the highest SMG/SDG ratios of 0.7 and 0.4. Kinetic analyses show that diglucosylation is an essentially irreversible reaction, while monoglycosylation is kinetically favored. The results lay the foundation for the biotechnological production of SMG.

## Introduction

Lignans, a type of diphenolic, nonsteroidal phytoestrogens found in many seeds, have many health benefits.^1−3^ Lignans from flaxseed usually occur in the form of ester cross-linked secoisolariciresinol (SECO) diglucosides (SDGs) and form a lignan macromolecule.^3^ The monomeric aglycone (SECO) and intermediate monoglucoside forms (SMG) do not accumulate in large amounts in plants. The macromolecular lignan complex is hydrolyzed after ingestion, and SDG is deglucosylated to SECO, which is then absorbed in the intestine. The microflora in the large intestine converts unabsorbed SECO, which accounts for 50–72% of ingested SECO, into enterolignans including enterolactone (ENL) and enterodiol (END).^3−5^ Currently, SECO and SMG are only obtained by acid hydrolysis of SDG.^6^ However, enzymatic hydrolysis and microbial biotransformation also provide SECO and SMG.^7−9^ Since deglucosylation of SDG is necessary for absorption or conversion to END and ENL in vivo, the development of a functional flaxseed food with high bioavailability requires seeds with altered SECO glucosylation in vivo. The creation of flaxseed lines with reduced lignan glucosylation in the form of SECO or SMG in planta would be beneficial.^10^ Homozygous nonsense flax mutants generated by ethylmethanesulfonate mutagenesis completely lacked SDG but did not accumulate SECO.^10^ This implies a feedback inhibition mechanism. Creating flaxseed lines with increased SMG content is one way to circumvent this phenomenon.

Glycosylation is an important physiological reaction catalyzed by nucleoside diphosphate sugar-dependent glycosyltransferases (GTs) that alters the physicochemical properties of small molecules such as water solubility, stability, volatility, bioactivity, and bioavailability.^11,12^ Among several enzyme families that can form glycoside bonds, uridine diphosphate (UDP)-dependent glycosyltransferases (UGTs) produce glycosides by transferring a sugar moiety from a UDP-sugar donor to an acceptor molecule via an SN2-like mechanism, resulting in inversion of the configuration of the anomeric carbon.^13−15^ Plant UGTs have a conserved 44 amino acid long motif at the C-terminus of their amino acid sequences, known as the PSPG box (plant secondary (specialized) product glycosyltransferase), which is why they are classified as family 1 glycosyltransferases (GT1) according to the CAZy database (http://www.cazy.org/GlycosylTransferases.html).^11^ In addition, they carry a catalytically active His in the N-terminus, invert the anomeric center during catalysis, and adopt the GT-B fold.^16^ UGTs are particularly involved in the glycosylation of numerous plant metabolites, including polyphenols, alkaloids, and terpenoids. Since the modification of secondary metabolites alters their toxicity, transport, and storage and increases protection against biotic and abiotic stress, UGTs promote plant growth and development.^17,18^ The translocation of glycosides of monolignols, such as 4-coumaryl, coniferyl, and sinapyl alcohols, into the cell wall is essential for their polymerization and thus for the biosynthesis of lignin.^19^ With advances in sequencing techniques and the advent of genome sequencing, the number of putative UGTs has multiplied, but only a small number of them has been thoroughly explored. The analysis of UGTs has the potential to uncover numerous enzymes that could be used for industrial applications. There has been a recent surge of interest in UGTs, which could enable the biotechnological production of physiologically active metabolites such as steviosides, cardiotonic steroids, and C-glycosides.^12,20,21^ In this context, LuUGT74S1 from flax (*Linum usitatissimum*) plays a role as the enzyme catalyzes the sequential glucosylation of SECO to SMG and further to SDG.^22,23^ LuUGT74S1 is a single-copy gene and an important regulator of SDG synthesis in flax.^10^

The aim of this study was to identify critical amino acids that are important for the enzymatic activity of LuUGT74S1 and especially the SMG/SDG product ratio. It was hypothesized that by reducing the binding pocket for the acceptor molecules, the activity and product ratio could be altered. Biochemical analysis of LuUGT74S1 mutants had already shown that His352 and Trp355 are critical amino acids for glucosylation, while Gln337 and Ser357 appear to be required for the conversion of SMG to SDG in vitro.^24^ These amino acids are located within the PSPG motif, the donor-binding site of LuUGT74S1 of flax. In the present study, SECO and SMG were docked into the active site of Alphafold-generated LuUGT74S1, and amino acids in the vicinity (5 Å) of the acceptor substrate-binding site were mutated to alter enzymatic activity and the ratio of the products SMG and SDG. The results can help to produce more bioavailable SMG in a biotechnological process in the near future.

## Materials and Methods

### Chemicals

All chemicals were purchased from Sigma-Aldrich, Merck (Darmstadt, Germany), Carl Roth (Karlsruhe, Germany), and Thermo Fisher Scientific (Dreieich, Germany) unless otherwise noted. Uridine-diphosphate glucose (UDP-glucose) was obtained from Sigma-Aldrich. All substrates used for enzymatic reactions, including those employed for substrate screening via liquid chromatography–mass spectrometry (LC–MS) and UDP Glo Glycosyltransferase Assay (Promega, Walldorf, Germany), were diluted in dimethyl sulfoxide (DMSO).

### Molecular Modeling and Docking

A 3D-structure homology model of LuUGT74S1 was produced using the IntFOLD 8 (https://www.reading.ac.uk/bioinf/IntFOLD/9)^25^ and AlphaFold (https://deepmind.google/technologies/alphafold/)^26^ servers. The Swissmodel server (https://swissmodel.expasy.org/interactive) uses the protein sequence entered to search for similar amino acid sequences whose crystal structures are deposited in the protein database (pdb). Proteins with known crystal structures were found that showed sequence identities of 46% with LuUGT74S1 (PDB: 6l90; PDB: 5u6n) and were used as templates to guide the modeling of the target protein. The model with the highest confidence score was uploaded into UCSF Chimera 1.15 (https://www.cgl.ucsf.edu/chimera) for visualization and comparative analysis.^27,28^ Ligand docking was performed with the AutoDock Vina tool implemented in UCSF Chimera 1.15.^29^ Binding energies (Δ*G*) calculated by UCSF Chimera 1.15 were used to calculate equilibrium dissociation constants *K*_D_ by *K*_d_ = e^–Δ*G*/*R*/*T*^ and *K*_D_ = *K*_d_*c with *R* = 1.986 cal/mol/K, *T* = 298.15 K, and the standard reference concentration *c* = 1 mol/L.

### Cloning of LuUGT74S1 and Production of the Mutant Proteins

Genewiz, Leipzig, Germany (http://www.genewiz.com), after codon optimization for *Escherichia coli* (Supporting Information Figure S1), synthesized LuUGT74S1. The gene was ligated into the pGEX-4T-1 vector using *Eco*RI at the 5′-end and the NotI site at the 3′-end. UGT74S1-Y144F, S115A, S115F, Q136E, H194S, Q136F, A17S, Q136S, S82F, and E189L primers were designed (Supporting Information Table S1) and used to generate the mutant proteins by site-directed mutagenesis using the QuickChange protocol (Agilent Technology, Santa Clara, CA). The temperature program included one cycle for 3 min at 95 °C, 30 cycles for 30 s at 95 °C, 30 s at 65 °C, and 9 min at 75 °C, 1 cycle for 10 min at 72 °C, and cooling at 4 °C, using the appropriate primers. After Dpn I digestion of the templates, the PCR products were transformed into *E. coli* NEB 10 beta, followed by colony PCR, agarose gel electrophoresis, and sequence confirmation (Supporting Information Figure S2).

### Protein Production

Protein expression was performed using *E. coli* BL21(DE3) pLysS cells transformed with pGEX-4T-1-*LuUGT74S1* or the corresponding mutant genes. After overnight preculture at 37 °C and 150 rpm in a Luria–Bertani medium containing 100 μg/mL ampicillin and 34 μg/mL chloramphenicol, 10 mL of the preculture was added to 1 L of the main culture containing the corresponding antibiotics and incubated at 37 °C and 120 rpm until the OD600 reached 1 in a Chicane flask. Gene expression was induced with 1 mM isopropyl-β-d-thiogalactopyranoside, and cultures were incubated overnight at 18 °C and 150 rpm. Cells were harvested by centrifugation and stored at −80 °C. Recombinant fusion proteins with an N-terminal GST tag were purified using Novagen GST Bind Resin (Merck, Darmstadt, Germany) according to the manufacturer’s instructions. After resuspension, the cells were disrupted by sonication. After centrifugation, the crude protein extract was incubated overnight at 4 °C with the resin to bind the GST fusion protein, which was eluted with a GST elution buffer containing reduced glutathione. The quality of the purified proteins was verified by SDS-PAGE (Supporting Information Figure S3), and the protein concentration was determined with Roti-Nanoquant (Carl Roth, Karlsruhe, Germany) in 96-well microtiter plates according to the manufacturer’s instructions. Absorption was measured at 450 and 590 nm using a CLARIOstar plate reader (BMG Labtech, Germany).

### UDP-Glo Glycosyltransferase Assay

The data for the calculation of the enzyme kinetics were determined using the UDP-Glo Glycosyltransferase Assay (Promega, Mannheim, Germany).^14^ Assays with LuUGT74S1 and its mutants were performed at 30 °C for 30 min in a 50 mM phosphate buffer (pH 8) containing 100 μM UDP-glucose, substrate (dissolved in DMSO), and 5 μg of purified protein, made up to 100 μL with water. The reaction was stopped by addition of 12.5 μL of 0.6 M HCl and further neutralization with 1 M Trizma base. Five microliters of the UGT reaction mixture were pipetted into a 384-well plate. The luminescence reaction was started by adding 5 μL of UDP-Glo detection reagent and incubating for 30 min in the dark. The luminescence signal was detected with a CLARIOstar plate reader.^14^ The calculation of kinetic data was performed with KaleidaGraph (https://www.synergy.com/; v4.5.4).

### LC–MS Analysis

An Agilent 6340 Ion Trap mass spectrometer (Agilent Technologies, Santa Clara, CA, USA) connected to an Agilent 1200 HPLC system equipped with a capillary pump and a diode array detector was utilized. Components were separated with a Phenomenex Luna C18(2) column (150 mm long × 2.0 mm inner diameter, particle size 5 μm, 100 A; Phenomenex, Aschaffenburg, Germany) that was held at 28 °C. LC was performed with the following binary gradient system: solvent A, water with 0.1% formic acid, and solvent B, and 100% methanol with 0.1% formic acid. The gradient program was as follows: 0–30 min, 100% A to 50% A/50% B; 30–35 min, 50% A/50% B to 100% B, hold for 15 min; 100% B to 100% A, in 5 min, and then hold for 10 min. The injection volume was 5 μL, and the flow rate was 0.2 mL/min. The ionization parameters were as follows: the voltage of the capillary was 3500 V and the end plate was set to −500 V. The capillary exit was 121 V, and the Octopole RF amplitude was 171 Vpp. The temperature of the dry gas (N_2_) was 330 °C at a flow of 9 L/min, and the nebulizer pressure was 30 psi. Tandem MS was carried out using helium as the collision gas (4 × 10^–6^ mbar) with a 1 V collision voltage. The scan range was from *m*/*z* 50 to 975. Spectra were acquired in positive and negative ionization modes, and target ions were fragmented in auto MS2 mode. Metabolites were identified by their retention times, mass spectra, and product ion spectra in comparison with the data determined for authentic reference materials. Relative metabolite quantification was performed using DATA_ANALYSIS v.4.0 (Build 234) and QUANT_ANALYSIS v.2.0 (Build 234) software (Bruker Daltonik GmbH, Bremen, Germany). The results were normalized to the internal standard. UGT reactions were performed in a final volume of 100 μL of 100 mM phosphate buffer (pH 8) containing 5 μg of purified recombinant protein, 1 mM UDP-Glc, and 600 μM substrate (SECO) dissolved in DMSO. The reaction mixture was incubated at 30 °C with constant shaking at 400 rpm overnight (16 h) or 30 h for the time course experiment (0.17 μM protein, 200 μM SECO, 1.67 mM UDPG, 50 mM phosphate buffer (pH 8), stopped with 100 μL of trifluoroacetic acid). After centrifugation, the supernatant was analyzed via LC–MS analysis.^30^ Products were identified using authentic reference materials (Supporting Information Table S2 and Figure S4).

### Kinetic Analysis of LuUGT74S1 and Its Mutants

The kinetic parameters of LuUGT74S1 and its mutants were determined using a range of concentrations for the sugar acceptor substrate (70–1650 μM SECO with constant 1.64 mM UDP-glucose concentration) under optimal conditions. A total of 80 μg of protein was used to determine the apparent *v*_max_ and *K*_m_ values for SECO. The *k*_cat_ value was determined by dividing *v*_max_ by the molar concentration of the enzyme. Kinetic data were obtained by fitting to the Michaelis–Menten equation using KaleidaGraph (Version 4.5.4 for Windows, Synergy Software, Reading, PA, USA. http://www.synergy.com). Kinetic rate constants were calculated by KinTeK Explorer v11.0.1 (KinTek Corporation, Snow Shoe, USA).

## Results

### Modeling of LuUGT74S1 and Molecular Docking with SECO and SMG

**Figure 1:**
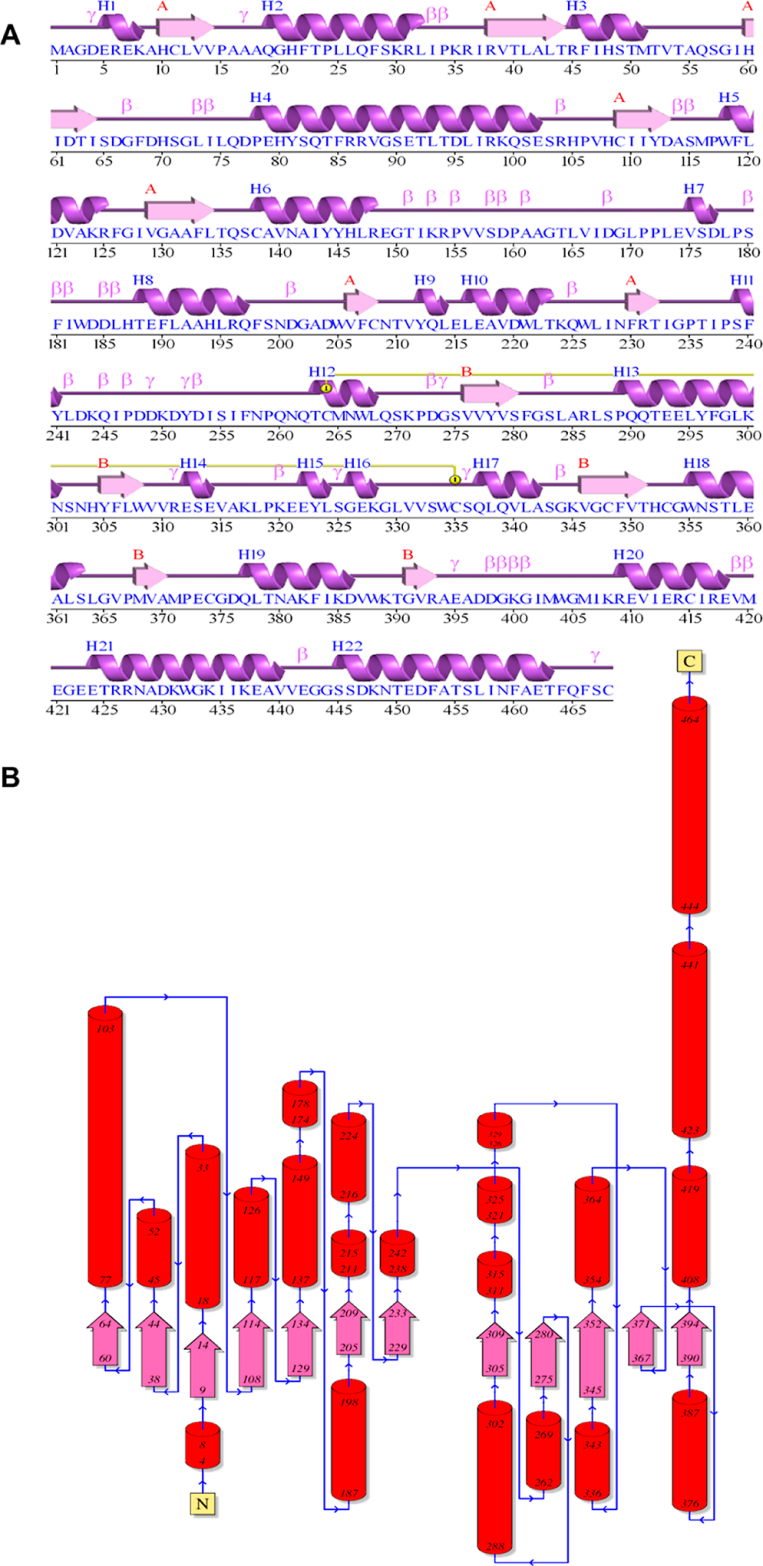
Predicted secondary structure of LuUGT74S1. (A) Schematic diagram showing the secondary structure elements of the protein (α-helices and β-sheets) together with various structural motifs such as β- and γ-turns. Helices are labeled H1, H2, etc., while strands are labeled A, B, C, etc. according to the β sheet to which they belong. (B) Topology of LuUGT74S1. Helices and sheets are shown as cylinders and arrows, respectively. N- and C-terminal ends are labeled. Both illustrations were generated by PDBsum (https://www.ebi.ac.uk/thornton-srv/databases/pdbsum/Generate.html).

LuUGT74S1 is unusual in that it glucosylates both symmetric hydroxyl groups in SECO, producing only trace amounts of the SMG intermediate, suggesting that both transferase reactions are equally favored.^22^ To determine the amino acids important for diglucosylation, PDBsum (https://www.ebi.ac.uk/thornton-srv/databases/pdbsum/Generate.html) was used to generate the 2D structure (Figure 1).

**Figure 2:**
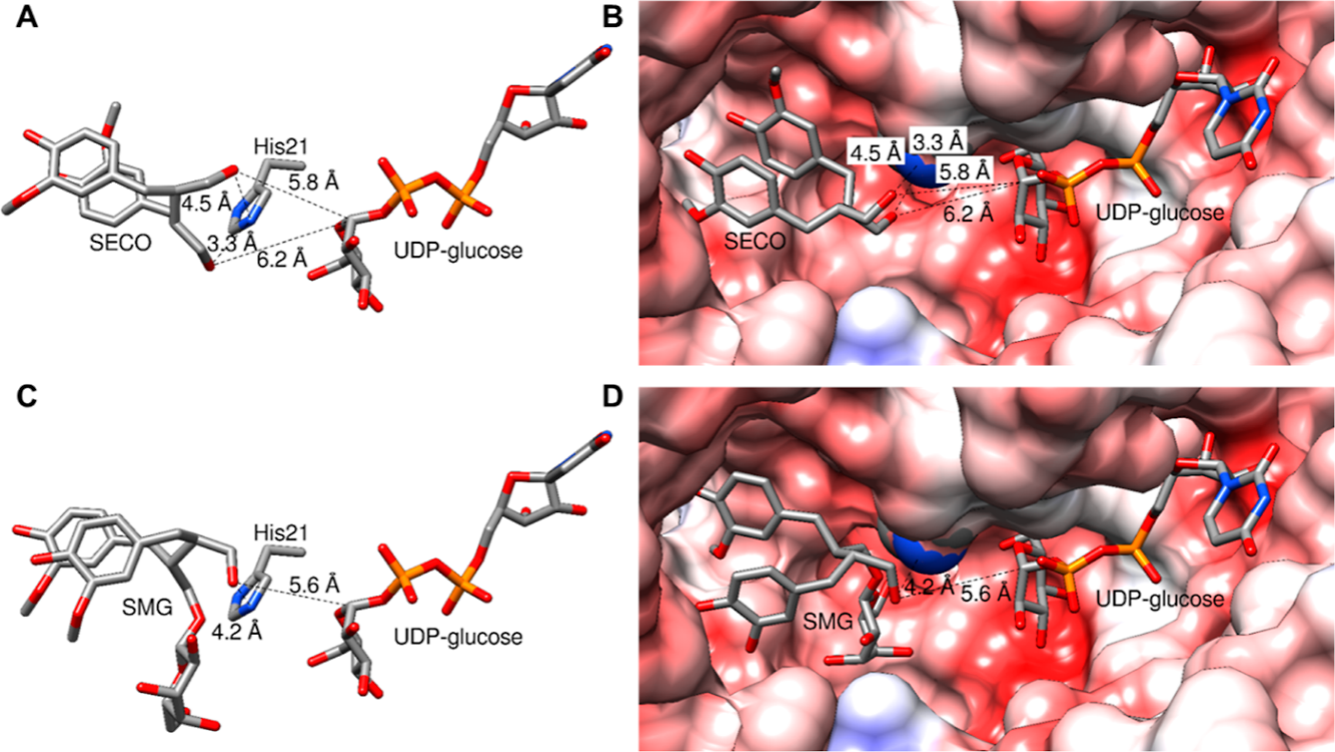
Arrangement of SECO and UDP-glucose in the active site of the predicted 3D structure of LuUGT74S1 by AlphaFold. (A) Stick representation of SECO, catalytically active His21, and acceptor substrate UDP-glucose. (B) Same representation as in (A), shown in the active-site pocket. (C) Stick representation of SMG, catalytically active His21, and UDP-glucose. (D) As in (C), shown in the active-site pocket.

**Figure 3:**
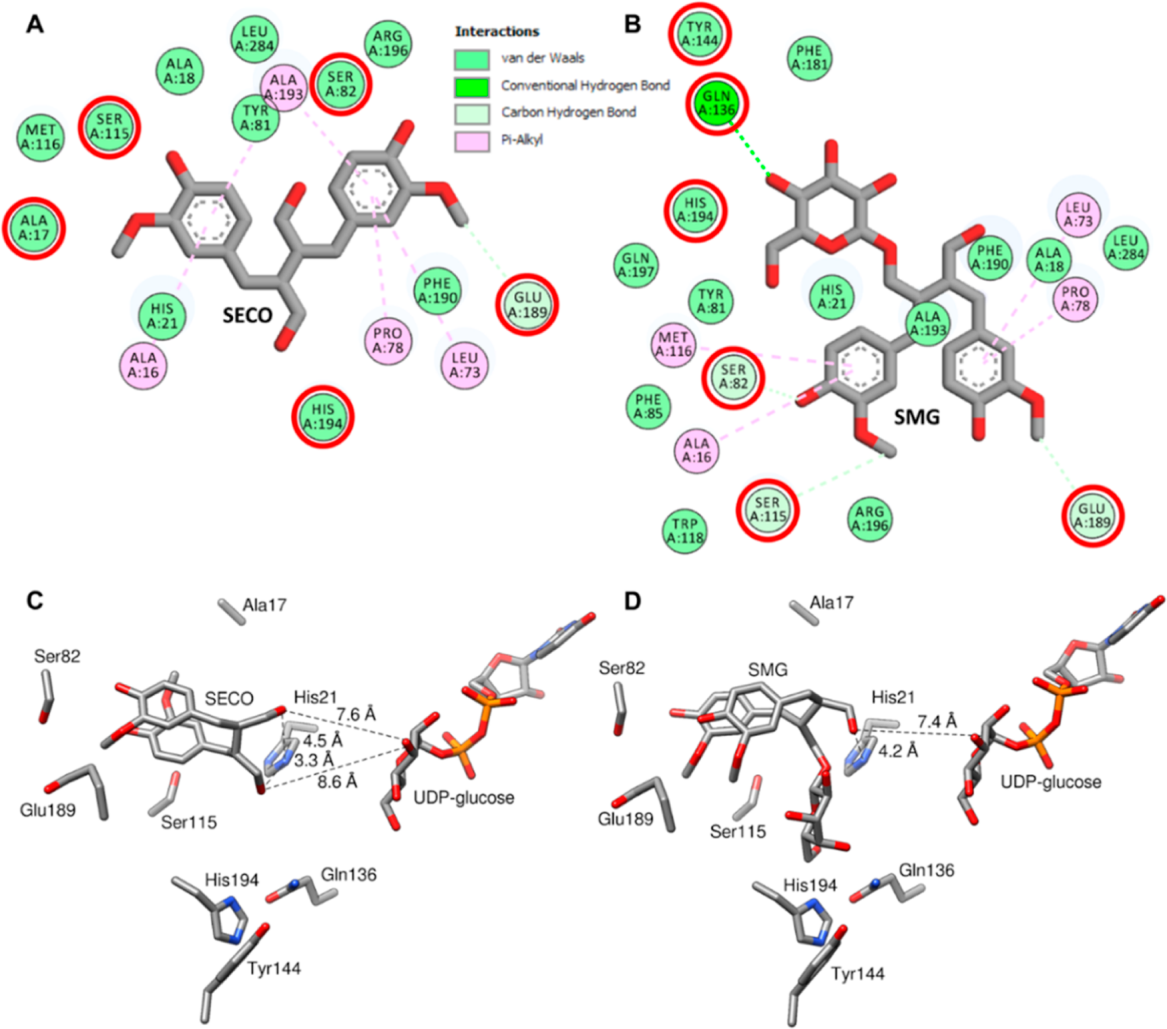
Amino acids at a distance of 5 Å from the acceptor substrates. (A) Amino acids in the vicinity of SECO. Those that have been mutated are marked with a red circle. (B) Amino acids in the vicinity of SMG. (C) 3D visualization of the mutated amino acids in the acceptor-binding pocket. SECO is shown. (D) Same as in (C), but SMG is shown.

LuUGT74S1 adopts a GT-B fold composed of two distinct N-terminal and C-terminal Rossmann-like domains of seven and five parallel β-sheets, respectively, linked to α-helices, connected by a linker region and an interdomain cleft. The conserved plant secondary product glycosyltransferase motif (PSPG) involved in donor substrate binding is located in the C-terminus at position Trp334 to Gln377, and the catalytically active His21 together with the activating Asp113 is located in the N-terminus (Figure 1). A disulfide bridge connecting Cys264 and Cys335 was predicted. The 3D structure was predicted using AlphaFold^26^ and InFOLD 8,^25^ and both SECO and SMG were fitted to the modeled active site using AutoDock Vina.^29^ The structures predicted by the two programs were similar to a root-mean-square distance (RMSD) of 2.167 Å, while most similar proteins with known crystal structures (sequence identities of 46% with LuUGT74S1; PDB: 6l90 and PDB: 5u6n) had RMSD values of 1.398 and 1.937 Å, respectively, compared to the structure of LuUGT74S1 generated by AlphaFold. Docking of the acceptor SECO to the active site of the lignan UGT showed that the phenyl residues of the substrate are arranged in parallel, and the two free primary hydroxyl groups point to the catalytically active histidine and the acceptor substrate (Figure 2). Interestingly, both reactive hydroxyl groups of the donor have similar distances to –Nε2 of His21 (3.3 and 4.5 Å) and –C1–H of UDP-glucose (5.8 and 6.2 Å). When SMG was docked to the predicted LuUGT74S1 structure, the SECO residue of SMG occupied the same space as the free SECO and the glucose moiety pointed downward into a cavity. The distance between free –O–H of SMG and –Nε2 of His21 is 4.2 Å, whereas the respective distance to –C1–H of UDP-glucose in the catalytic complex is 5.6 Å. The calculation of the dissociation constants *K*_D_ for SECO and SMG from the Δ*G* values of −6.1 and −7.9 kcal/mol obtained from the in silico docking experiments yielded values of 33.6 and 1.6 μM, respectively. SMG thus appears to have a higher affinity for LuUGT74S1 than SECO. In the 5 Å distance around the acceptor substrates SECO and SMG in the predicted 3D model of LuUGT74S1, there are 16 and 21 amino acid residues, respectively, including His21 (Figure 3). They interact with the acceptors via van der Waals bonds, carbon–hydrogen bonds, and π-alkyl and conventional hydrogen bonds. To experimentally analyze the significance of the different amino acids for LuUGT74S1 catalysis and the ratio of the products, seven were exchanged and 10 mutant proteins were generated. The amino acids Ala17, Ser82, Ser115, and Glu189 were selected because they form the binding pocket for the guaiacol residues of the acceptors, while Gln136, Tyr144, and His194 interact with the glucose residue of SMG. The exchange of amino acids in the binding site for the 2-methoxyphenol residues should primarily affect catalysis, while changes in the binding pocket of the glucose residue should alter the product ratio.

### Analysis of UGT74S1 Mutations

**Figure 4:**
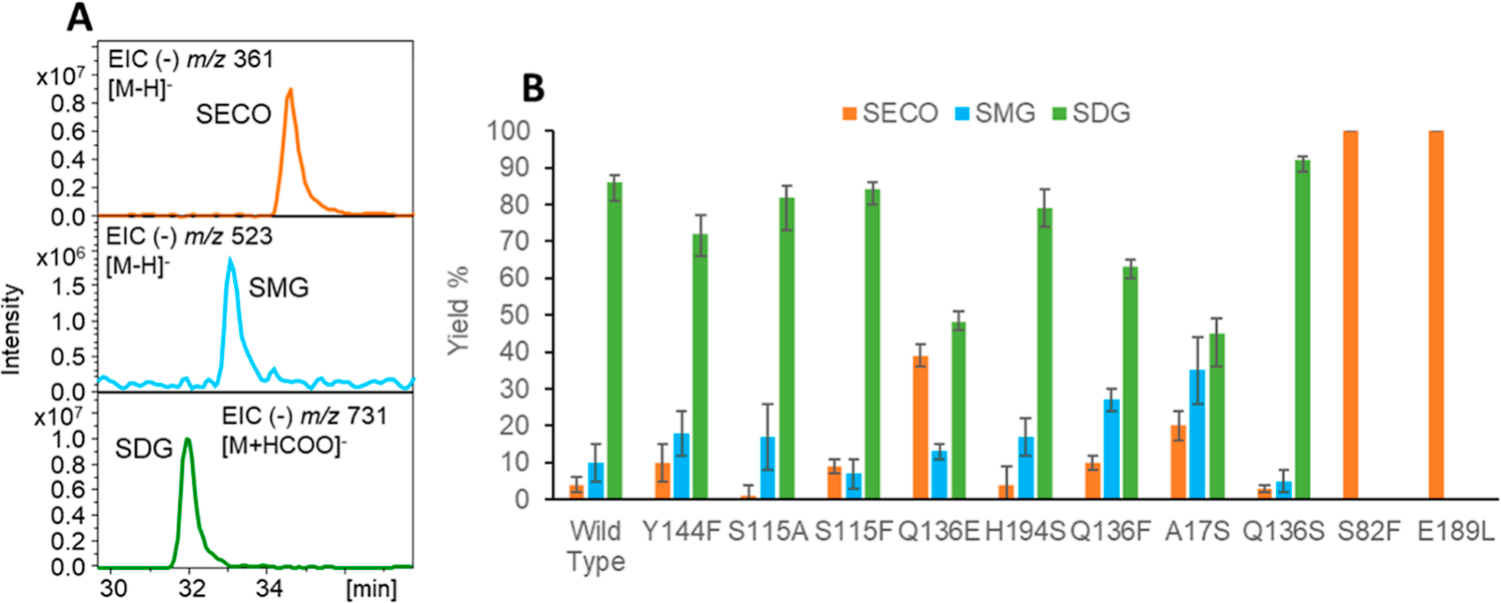
LC/MS analysis of LuUGT74S1 mutants. (A) LC/MS enabled the quantification of SECO, SMG, and SDG at ion traces in the negative mode of *m*/*z* 361, 523, and 731, respectively. (B) Balancing of product yields. Three replicates were used to calculate the yields. Error bars are standard deviations from the mean. Amino acid codes with a letter indicate mutant proteins.

The LuUGT74S1 mutant genes encode the following amino acid substitutions: A17S, S82F, S115F, S115A, Q136S, Q136E, Q136F, Y144F, E189L, and H194S were generated by cloning, and agarose gel separation (Supporting Information Figure S2) and Sanger sequencing verified the sequences. To evaluate the expression and functionality of the different mutant proteins, the wild-type LuUGT74S1 and its 10 mutants were expressed in *E. coli*, and the proteins were purified and tested for identity by SDS-PAGE (Supporting Information Figure S3). Enzyme assays were performed with the purified proteins overnight (16 h), and product formation was quantified by LC–MS to determine the effects of changes in ligand binding sites generated by site-directed mutagenesis (Figure 4; Supporting Information Figure S4). The wild-type enzyme glucosylated SECO almost completely (4% remained) to SDG without substantial formation of the intermediate SMG (10%). The S115F mutant produced a similarly high amount of SDG (84%) as only 9% SECO and 7% SMG remained, while S115A produced higher levels of SMG (17%) and retained lower amounts of the substrate SECO (1%). The glucosylation of SECO by the mutants Y144F, H194S, Q136E, and Q136F resulted in lower SDG amounts than the wild type (72, 79, 48, and 63%, respectively), with a concomitant increase in the concentration of the intermediate SMG (18, 17, 13, and 27%, respectively).

This was most pronounced for mutant A17S with 20 and 35% relative yields for SECO and SMG, respectively. While the mutants S82F and E189L were inactive, Q136S showed the highest activity, which was even higher than that of the wild type, as SECO was almost completely converted (3% remained), and 92% of SDG was produced. It appears that mutations in the predicted binding pocket of the glucose part of SMG (positions 136, 144, and 194) reduce the total catalytic activity of LuUGT74S1, while substitutions from polar to nonpolar amino acids in the binding pocket of the guaiacol part of SECO and SMG (positions 82 and 189) completely abolish the enzymatic activity (Figure 3). The reciprocal exchange of A/S and S/A at positions 17 and 115, respectively, amino acids encompassing SECO and SMG, decreased glucosylation activity for A17S but not for S115A. Polar and charged amino acids such as S82 and E189 interact with the substituents of the guaiacol ring systems and are essential for catalytic activity, probably due to stabilizing the correct orientation of the substrates. An exchange with nonpolar amino acids (S82F and E189L) therefore renders the enzyme inactive (Figure 3).

### Enzyme Kinetics of SECO and Its Mutant

Kinetic parameters were determined by the UDP-Glo assay, which quantifies the amount of UDP formed by the enzyme with enzyme assays running for 30 min (Table 1; Supporting Information Figure S5).

**Table 1:** Kinetic Parameters of Wild Type and Mutant LuUGT74S1 toward SECO^a^

	*K*_m_ [μM]	*v*_max_ [nkat mg^–1^]	*k*_cat_ [s^–1^]	*k*_cat_/*K*_m_ [mM^–1^ s^–1^]
WILD	74 ± 1	15.8 ± 1.7	0.80 ± 0.01	11
Y144F	72 ± 1	15.0 ± 1.0	0.75 ± 0.02	10
S115A	70 ± 2	14.9 ± 1.1	0.76 ± 0.08	11
S115F	75 ± 1	13.9 ± 0.5	0.77 ± 0.02	10
Q136E	69 ± 2	14.3 ± 1.3	0.75 ± 0.02	11
Q136S	75 ± 1	17.0 ± 0.5	0.92 ± 0.71	12
Q136F	165 ± 9	5.4 ± 1.0	0.29 ± 0.04	2
H194S	122 ± 12	6.4 ± 0.7	0.34 ± 0.06	3
A17S	68 ± 1	12.5 ± 1.1	0.56 ± 0.45	8

a *v*_max_—the maximal reaction rate; *K*_m_—Michaelis–Menten constant; *k*_cat_ [s^–1^]—catalytic rate. *k*_cat_/*K*_m_ [mM^–1^ s^–1^]—catalytic efficiency.

Wild-type LuUGT74S1 and mutant proteins Y144F, S115A, S115F, Q136E, and Q136E showed *K*_m_ and *v*_max_ values that were not significantly different. However, higher *K*_m_ and lower *v*_max_ values were determined for Q136F and H194S than the wild-type enzyme, and A17S showed a slightly reduced *K*_m_ and *v*_max_ value. Overall, the effects of some mutations on the kinetic parameters were moderate, indicating a certain degree of flexibility of the amino acids in the active site. Q136S even outperformed LuUGT74S1 in terms of catalytic efficiency (Table 1). Since the UDP release is quantified for the calculation of *K*_m_ and *v*_max_, the values must be interpreted with caution, as the mono- and diglucosylation take place directly after each other and the individual conversions cannot be distinguished. The values, therefore, refer to the total catalytic activity.

### Time Course Experiment

Since in most cases the intermediate SMG was only detected in small amounts after termination of the reaction, we performed a time course experiment over 30 h with an extremely low enzyme concentration (0.17 μM) to investigate the formation kinetics of SMG.

**Figure 5:**
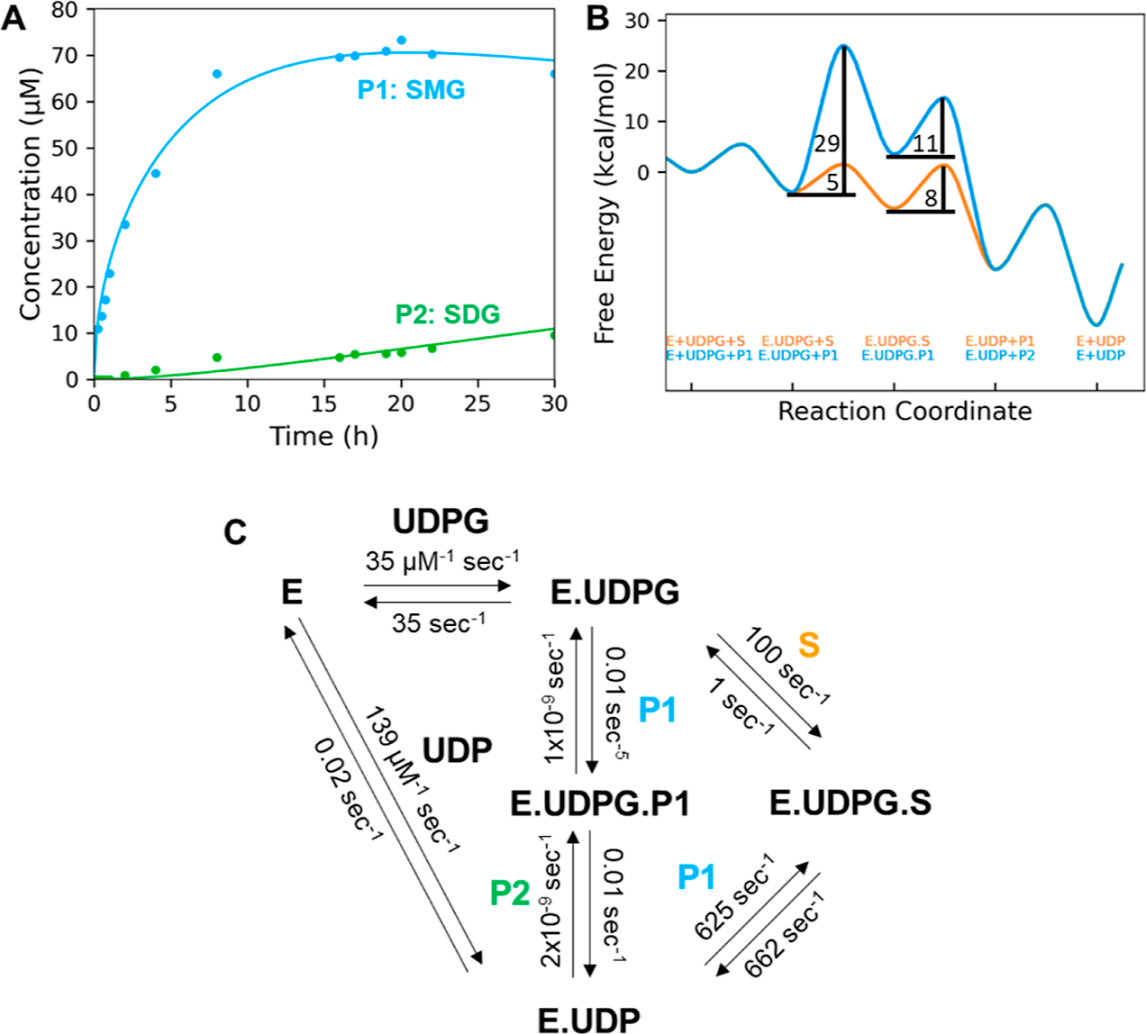
Time course experiment to calculate reaction rates with KinTec explorer. (A) LuUGT74S1 (0.17 μM) was used to produce SMG (P1) and SDG (P2) from 200 μM SECO for 30 h. Products were quantified by LC–MS. (B) Free energy profile calculated by KinTek Explorer. (C) Reaction scheme and calculated reaction rates. E, enzyme; S, SECO; P1, SMG; P2, SDG, and complexes thereof.

The products SMG and SDG were quantified by LC–MS, and the data was fitted with KinTek Explorer to obtain velocity constants and the free energy profile (Figure 5). Although the quantification of the reactants by LC–MS showed some variability, the kinetic data of the exothermic reaction could be calculated. While the rate constant for the forward reaction of SECO (S) binding is about 100 times the reverse reaction (100 and 1 s^–1^) and much higher than the forward reaction rate for SMG (P1) binding (0.01 s^–1^), the reaction rate for reverse SMG binding (1 × 10^–9^ s^–1^) is negligibly small, making this reaction irreversible. The higher value for the reverse reaction rate of UDP release from the enzyme (139 μM^–1^ s^–1^) compared to the forward reaction (0.02 s^–1^) confirms the product inhibition of UDP known for many UGTs.^31^ The high activation energy of about 29 kcal/mol for the formation of the enzyme–UDPG-P1(SMG) complex explains the low reaction rate of 0.01 s^–1^, which leads to the formation of the complex (Figure 5). The activation energy for the formation of the enzyme–UDPG–SECO complex is only 5 kcal/mol. While the release of SMG (P1) is the rate-limiting step for monoglucosylation (8 kcal/mol), the formation of the E.UDPG.P1 complex (29 kcal/mol) is the bottleneck for diglucosylation.

## Discussion

In this study, molecular docking, site-directed mutagenesis, and enzyme activity assays were performed to investigate the role of amino acid residues in the active site of LuUGT74S1 from flax for catalysis. Larger amounts of intermediate SMG should be formed by mutation in the predicted acceptor-binding site of the two-stage glycosylation enzyme. After constructing the 3D structure of the LuUGT47S1 protein, the binding sites of both substrates were predicted in silico, and various mutants with altered amino acids in the active site were experimentally studied.

**Figure 6:**
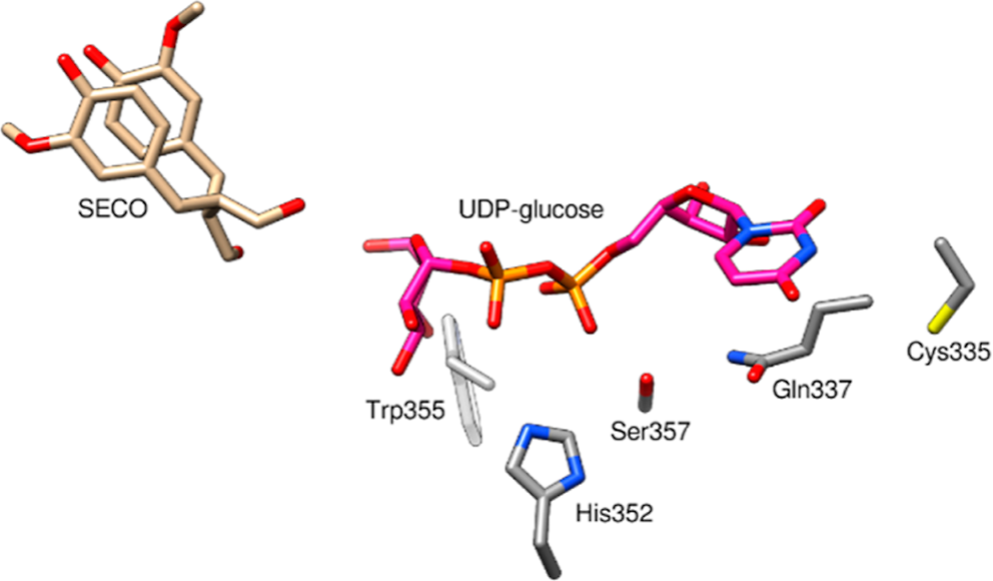
Amino acids of LuUGT74S1 were mutated in a previous report. Cys335, Gln337, His 352, Ser357, and Trp355 that shape the UDP-glucose binding site in LuUGT74S1 were mutated, and the mutant enzymes were experimentally analyzed.^24^

In a previous study, LuUGT74S1 mutants C335A, Q337A, H352D, S357A, W355A, and W355G were analyzed.^24^ The corresponding amino acids are part of the highly conserved PSPG box, residues of which are responsible for binding of the donor substrate UDP-glucose (Figure 6).^11^ A significant reduction in GT activity was observed for all mutants, while W355A, W355G, and H352D were completely inactive. C335A, Q337A, and S357A showed about 20, 3, and 3% of the catalytic activity of LuUGT74S1 toward SECO, respectively. The C335A mutant was found to have a significantly 4-fold increased concentration of SMG compared with that of the wild-type enzyme. In the present study, a similar increase was achieved with the A17S mutant (Figure 4).

We have analyzed the role of amino acids that constitute the binding site for the acceptor substrate by LC–MS (Figure 3; Supporting Information Figure S4). The protein expression patterns of the mutants in *E. coli* showed no significant differences (Supporting Information Figure S3). LuUGT74S1 and its mutants could thus be successfully expressed in *E. coli* as glutathione-*S*-transferase (GST) fusion proteins, whereas in the original publication, His6 fusion proteins were produced in yeast.^22^ In our mutants, the effects on catalytic activity were less pronounced compared to the effects of amino acid replacement in the donor-binding site, although two inactive mutants (S82F and E189L) were also obtained (Supporting Information Figure S4). It is assumed that S82 and E189 would interact with the side chains of the guaiacol residue of SECO, and thus, the exchange affects catalysis.

When the small polar amino acid Ser was replaced by the large nonpolar Phe in position 115, the product spectrum was not dissimilar to the wild type (Supporting Information Figure S4), whereas more SMG (17%) remained in the assay with S115A (Figure 4). The role of Q136 in the enzymatic activity of LuUGT74S1 was more obvious as the exchange with the nonpolar Phe and especially the charged Glu reduced the activity, which was confirmed by the larger amount of remaining SECO compared to the wild type. The substitution of Q136 by the small polar Ser increased the activity as the highest relative amount of SDG (92%) was obtained (Supporting Information Figure S4). There is an obvious trend indicating that mutants with reduced transferase activity (Y144F, S114A, Q136E, Q136F, H194S, and A17S), as inferred from the large amount of unconsumed SECO after conversion (Supporting Information Figure S4), tend to form more SMG. This is particularly noticeable in this study with the mutants C335A^24^ and A17S. The C335A mutant showed a ratio of SMG/SDG of about 4, and A17S exhibited a ratio of 0.7 (Figure 4). While the interaction with the donor substrate should be impaired in the C335A mutant, the binding of the acceptor substrate should be affected in the A17S mutant.

The determination of Michaelis–Menten parameters of LuUGT74S1 and its mutants with the UDP-Glo assay (30 min incubation) showed that Y144F, S115A, S115F, and Q136E have comparable values to the wild type, while H194S, Q136F, and A17S showed reduced catalytic efficiency (*k*_cat_/*K*_m_) and Q136S even increased *k*_cat_/*K*_m_ values (Table 1). LC–MS analysis of overnight incubations with S115F and Q136S confirmed almost complete conversion of SECO to SDG, comparable to the results for the wild-type enzyme (Supporting Information Figure S4) and the kinetic data (Table 1). Mutants Y144F, S115A, and Q136E showed similar kinetic parameters to LuUGT74S1 (Table 1), but LC–MS analysis after 16 h of incubation still showed significant amounts of the substrate (Supporting Information Figure S4). It appears that the stability of the enzymes was affected by the mutations and that the proteins lost activity during the entire 16 h period. This would also explain the increased SMG levels produced by these mutants. The reduced catalytic efficiency of the mutants Q136F, H194S, and A17S (Table 1) is likely responsible for the SECO still being present after overnight incubation with these mutants (Supporting Information Figure S4). The SMG concentration produced by the mutants was low and appeared to increase in less catalytically active mutants, implying that a reduction in overall activity may lead to accumulation of the intermediate (Table 1). Since the UDP-Glo assay quantifies the UDP released from the overall reaction, it is difficult to say which partial reaction is affected, but we hypothesize that mono- and diglucosylation activity is reduced in mutants A17S and Q136F, respectively. As the obtained SMG content is still low, multiple mutations should be introduced into the enzyme in the future to further reduce its catalytic activity toward SMG.

Finally, we modeled the two-step glycosylation reaction and calculated the reaction rates with KinTek Explorer (Figure 5). Although the monoglucosylation reaction is kinetically favored, diglucosylation predominates because the equilibrium constants *K*_eq_ = *k*_forward_/*k*_reverse_ for the formation of the enzyme–UDPG-S (*K*_eq_ = 100) and enzyme–UDP complex (*K*_eq_ = 1) are significantly smaller than the corresponding values for the formation of the enzyme–UDPG-P1 (*K*_eq_ = 1 × 10^7^) and E-UDP complex (*K*_eq_ = 5 × 10^6^). The equilibrium therefore eventually shifts to SDG. This indicates that diglucosylation is an essentially irreversible reaction. Since SMG (P1) binding is already disfavored compared to SECO binding, this explains why mutations in the SMG binding pocket led to only a moderate increase in SMG concentration.

Flax LuUGT74S1 converts SECO into SMG and SDG in a sequential process. Since SMG does not accumulate in plants but is more bioavailable and not commercially available, an attempt was made to generate LuUGT74S1 variants that form more SMG. For this purpose, amino acids were selected based on a predicted 3D structural model that interact with the sugar and guaiacol moiety of SMG in the binding pocket of the acceptor. While mutations of amino acids that interact with the acceptor’s aromatic ring system (S82F and E189L) rendered the protein catalytically inactive (Figure 3; Figure 4), positions 17 and 136 appear to be important for diglucosylation. This can be clearly deduced from the fact that mutants Q136F and A17S produced 27 and 35% SMG, respectively, compared to 10% SMG of the wild-type enzyme (Figure 4). The change in the size and polarity of the amino acid side chains in the mutants resulted in reduced diglucosylation activity. The strategy of reducing the size of the cavity that interacts with the glucose portion of SMG appears to contribute to increasing the SMG content (Figure 3). However, the results also show that multiple mutations are necessary to obtain significant amounts of SMG by rational design.

Surprisingly, LuUGT74S1 knockout plants incapable of producing SDG accumulated low levels of SMG but not SECO.^10^ This finding suggests that two paralogs of LuUGT74S1, namely, LuUGT74S3 and LuUGT74S4, which cannot form SDG but glucosylate SECO to SMG,^23^ can produce trace amounts of SMG. Thus, new flax varieties with SMG as a new trait are available, but due to the low levels of the metabolite, there is still room for improvement.^10^

## Supporting Information Available

Forward and reverse primers used for site-directed mutagenesis of LuUGT74S1; mass-to-charge ratios *m*/*z* of the substrate and the glucoside products used for LC–MS detection; original and optimized (for *E. coli*) gene sequence of LuUGT74S1 (from flax seed) and the protein sequence; control of the size of the ligated PCR products of the mutated LuUGT74S1 genes by agarose gel electrophoresis; SDS-PAGE analysis to separate and identify protein sizes of LuUGT74S1 wild type and mutations; LC/MS analysis of the in vitro enzyme activity of all mutant proteins with the exception of S82F and E189L; and Michaelis–Menten diagrams of mutant proteins (PDF)
