# Supplementary material for: Kinetics of Secoisolariciresinol Glucosyltransferase LuUGT74S1 and Its Mutants
Source: J Agric Food Chem. 2024 Aug 30;72(36):20005–13. doi: 10.1021/acs.jafc.4c06229 (PMC11403609; doi:10.1021/acs.jafc.4c06229)
Supplement: Supplementary file 1 — jf4c06229_si_001.pdf [file jf4c06229_si_001.pdf]

SUPPLEMENTARY INFORMATION

Kinetics of the secoisolariciresinol glucosyltransferase LuUGT74S1 and its mutants

Sadiq Saleh Moree [1,2], Lukas Böhm[1], Thomas Hoffmann[1] Wilfried G. Schwab[1*]

[1] Biotechnology of Natural Products, Technische Universität München, Liesel-Beckmann-Str. 1, 85354 Freising, Germany;
[2] Department of Biochemistry, University of Thamar, P.O. Box 87246, Sana’a-Tiaz Road, Thamar, Yemen.

Table S1. Forward and Reverse Primers used for site-directed mutagenesis of *LuUGT74S1*.

	Primer ID	Direction	Primer sequence (5'-3')
**1**	Y144F	F	GCGGTGAACGCGATTTTTATCATCTGCGCGAAGGC
		R	GCCTTCGCGCAGATGATAAAAATCGCGTTCACCGC
**2**	S115A	F	TTATTTATGATGCGGCCATGCCGTGGTTTCTGGATG
		R	CATCCAGAAACCACGGCATGGCCGCATCATAAATAA
**3**	S115F	F	TTATTTATGATGCGTTTATGCCGTGGTTTCTGGATG
		R	CATCCAGAAACCACGGCATAAACGCATCATAAATAA
**4**	Q136E	F	CGCGGCGTTTCTGACGGAGAGCTGCGCGGTGAACG
		R	CGTTCACCGCGCAGCTCTCCGTCAGAAACGCCGCG
**5**	H194S	F	AATTTCTGGCGGCGTCTCTGCGTCAGTTTAGCAACG
		R	CGTTGCTAAACTGACGCAGAGACGCCGCCAGAAATT
**6**	Q136F	F	GCGCGGCGTTTCTGACGTTTAGCTGCGCGGTGAACG
		R	CGTTCACCGCGCAGCTAAACGTCAGAAACGCCGCGC
**7**	A17S	F	CCTGGTGGTGCCGGCGAGCGCGCAAGGCCATTTTAC
		R	GTAAAATGGCCTTGCGCGCTCGCCGGCACCACCAGG
**8**	Q136S	F	GCGGCGTTTCTGACGTCAAGCTGCGCGGTGAACGCG
		R	CGCGTTCACCGCGCAGCTTGACGTCAGAAACGCCGC
**9**	S82F	F	AAGATCCGGAACATTATTTTCAGACCTTTCGCCGCG
		R	CGCGGCGAAAGGTCTGAAAATAATGTTCCGGATCTT
**10**	E189L	F	GATGATCTGCATACCTTATTTCTGGCGGCGCATCTG
		R	CAGATGCGCCGCCAGAAATAAGGTATGCAGATCATC

Gene specific primers, 100% complementary, No overhangs

Table S2. The mass to charge ratios *m/z* of the substrate and the glucoside products used for LC-MS detection. M = molecule; H = proton; $HCOO^-$= formate, Cl^- = chloride.

Substrat/ product	M [g/mol]	$[M-H]^-$ [*m/z*]	$[M+CHOO]^-$ [*m/z*]	$[M+Cl]^-$ [*m/z*]
SECO	362	361	407	397
SMG	524	523	569	559
SDG	686	685	731	721

A

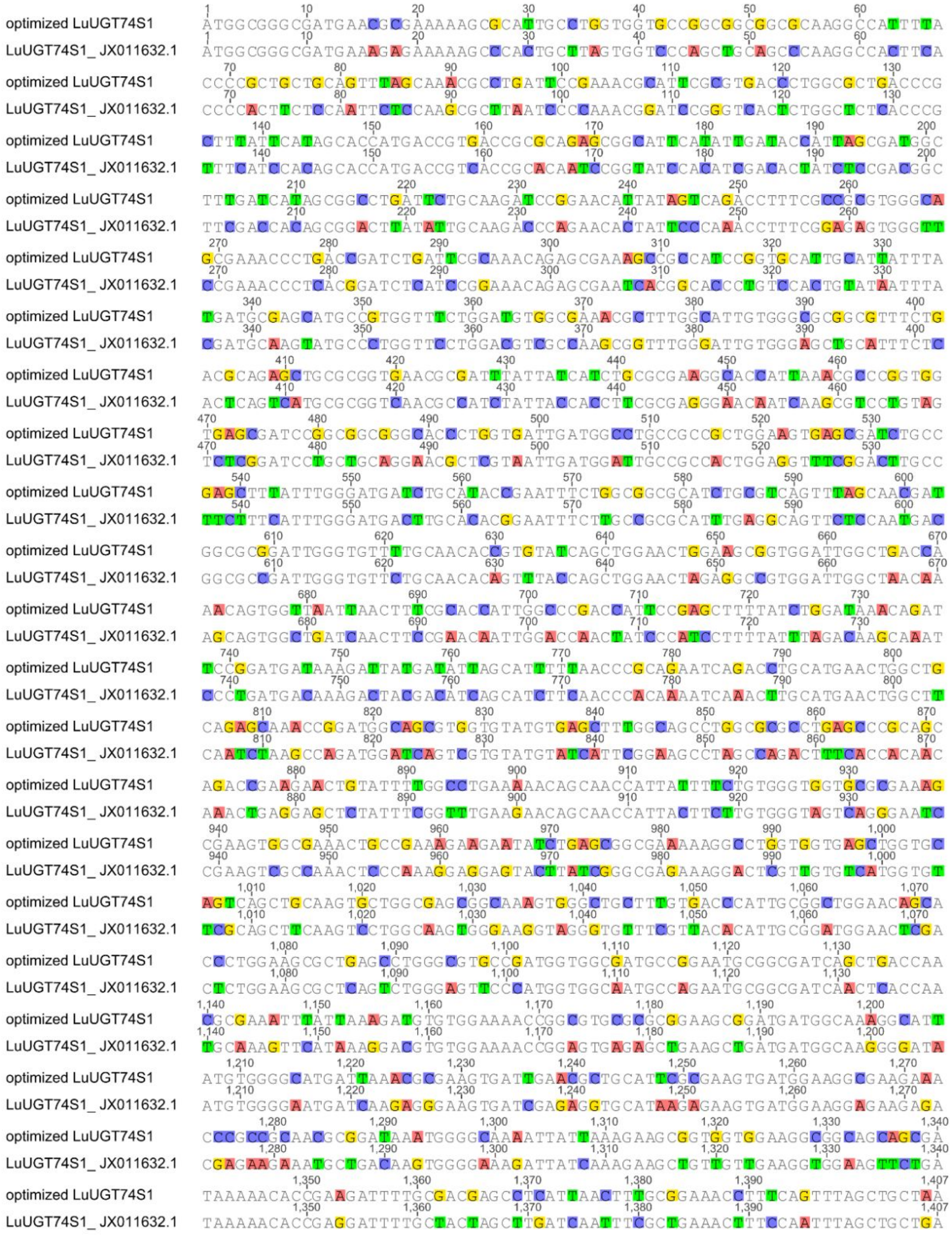

B

MAGDEREKAHCLVVPAAAQGHFTPLLQFSKRLIPKRIRVTLALTRFIHSTMTVTAQSGIHIDTISDGFDHSGLILQDPEHYSQTFRR
VGSETLTDLIRKQSESRHPVHCIIYDASMPWFLDVAKRFGIVGAAFLTQSCAVNAIYYHLREGTIKRPVVSDPAAGTLVIDGLPPLE
VSDLPSFIWDDLHTEFLAAHLRQFSNDGADWVFCNTVYQLELEAVDWLTKQWLINFRTIGPTIPSFYLDKQIPDDKDYDISIFNPQ
NQTCMNWLQSKPDGSVVYVSFGSLARLSPQQTEELYFGLKNSNHYFLWVVRESEVAKLPKEEYLSGEKGLVVSWCSQLQVLA
SGKVGCFVTHCGWNSTLEALSLGVPMVAMPECGDQLTNAKFIKDVWKTGVRAEADDGKGIMWGMIKREVIERCIREVMEGEET
RRNADKWGKIIKEAVVEGGSSDKNTEDFATSLINFAETFQFSC

Figure S1. The original and optimized (for *Escherichia coli*) gene sequence of *LuUGT74S1* (from Flax seed) and the protein sequence. (**A**) Nucleotide sequence of the optimized and original *LuUGT74S1* gene sequence. The deviating nucleotides are highlighted by individual colors. (**B**) The amino acid sequence of LuUGT74S1 derived from both sequences.

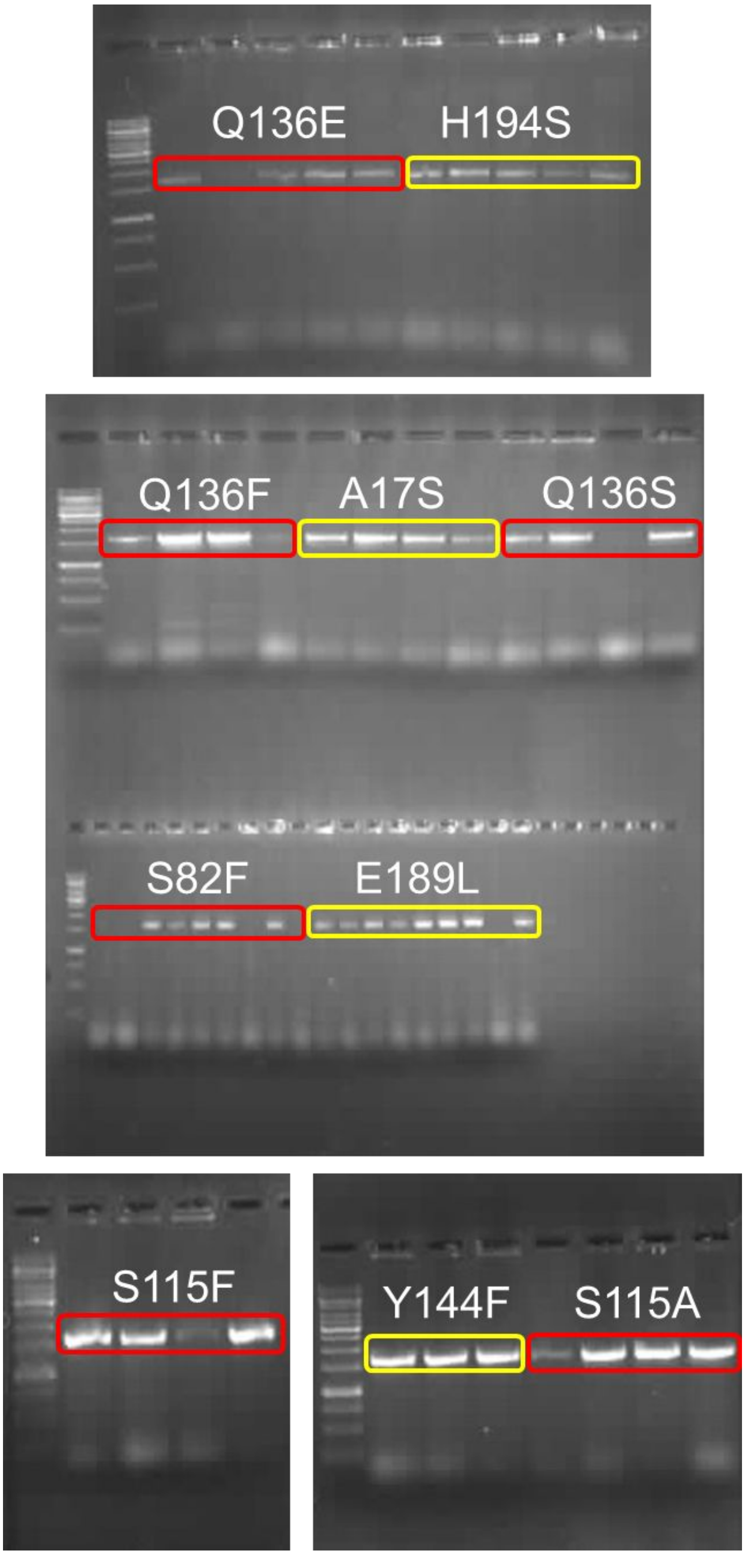

Figure S2. Control of the size of the ligated PCR products of the mutated *LuUGT74S1* genes by agrose gel electrophoresis. Colony PCR of the ten *LuUGT74S1* mutants. The boxes confirm the sizes of the mutant genes isolated from different colonies. For comparison, a 1 kb DNA ladder is shown on the left.

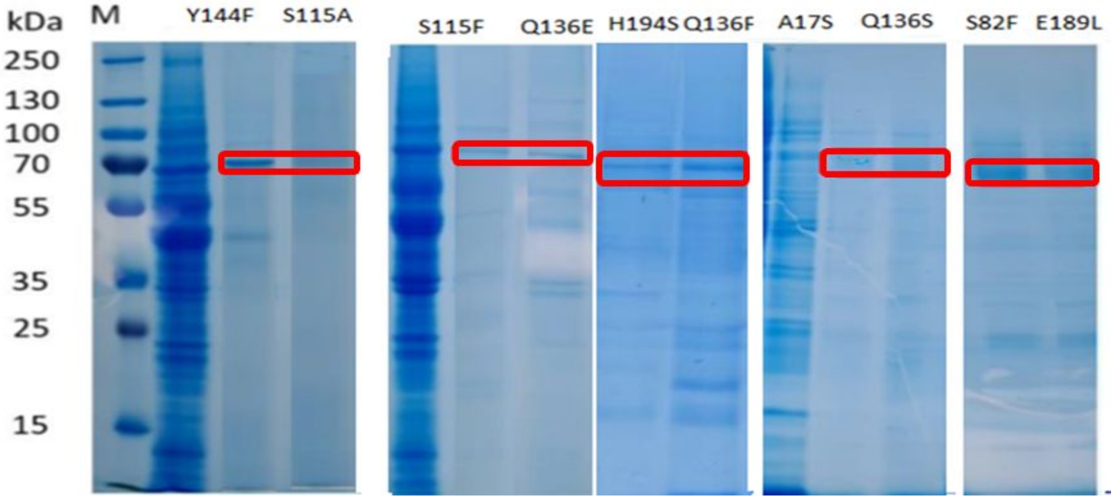

Figure S3. SDS-PAGE analysis to separate and identify protein sizes of LuUGT74S1 wild type and mutations. The bands framed in red represent proteins with a size of about 73 kDa (LuUGT74S1 and its mutants with 47 kDa, bound to GST with 26 kDa), as shown by the protein ladder on the left. All bands with red frame have the same size.

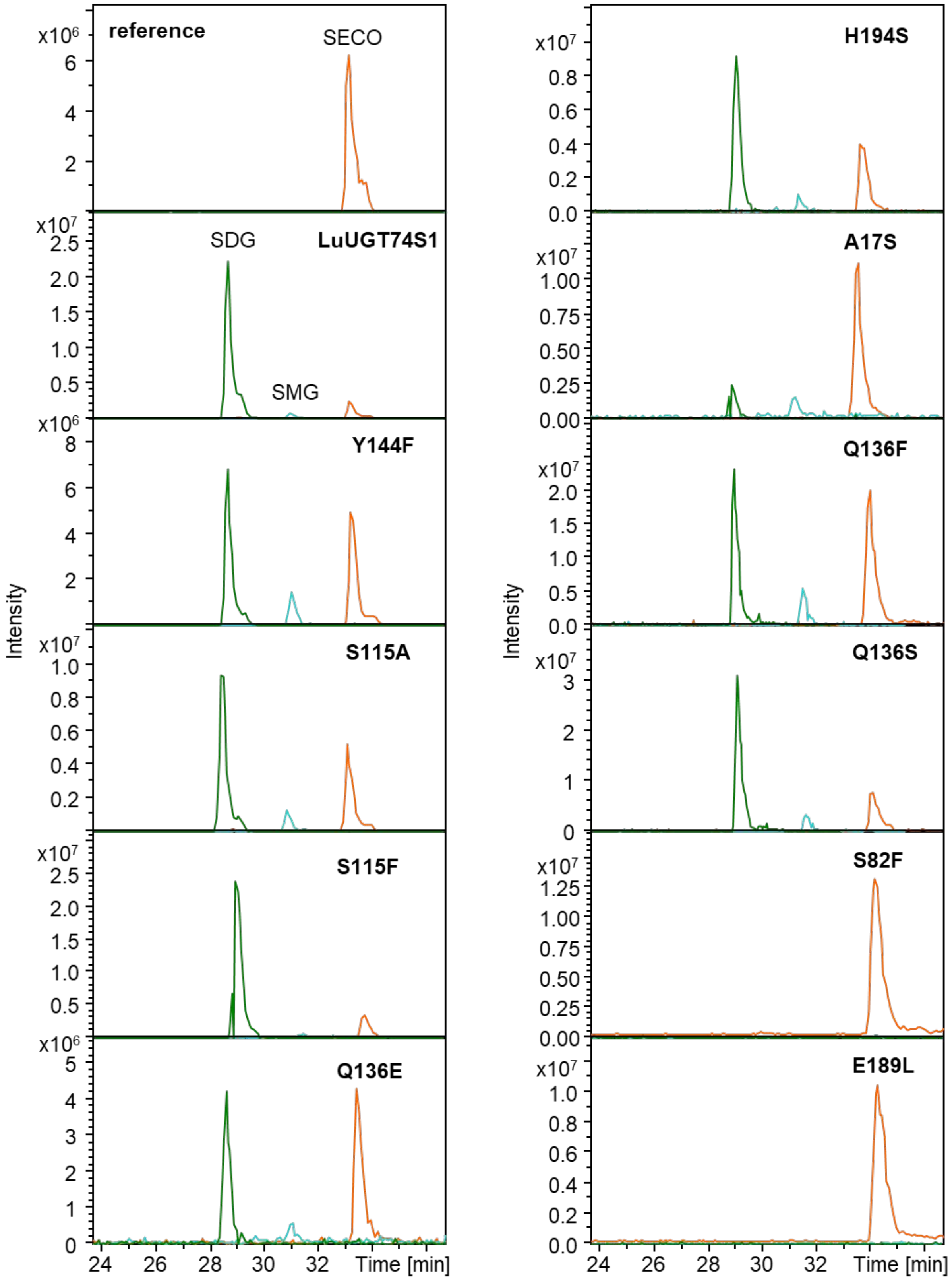

Figure S4. LC/MS analysis of the reaction products of the wild-type enzyme and all mutant proteins. Note that S82F and E189L do not produce SMG or SDG. Ion trace *m/z* 361 (orange) shows SECO, *m/z* 523 (light blue) shows SMG, and *m/z* 731 (green) shows SDG.

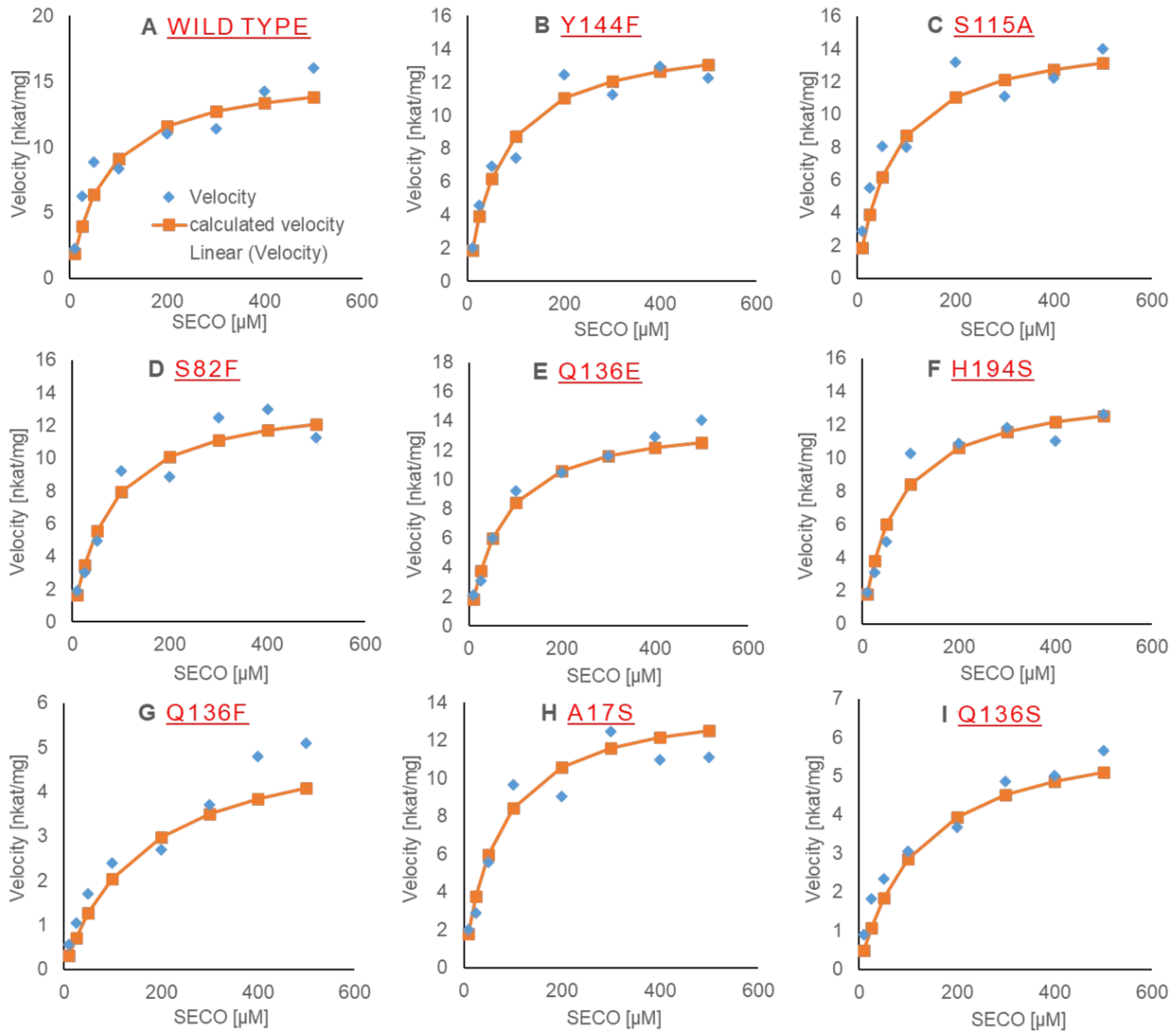

Figure S5. Michaelis-Menten diagrams of mutant proteins. Plots of acceptor substrate concentration versus velocity. **(A)** wild type**,** **(B)** Y144F, **(C)** S115A, **(D)** S82F, **(E)** Q136E, **(F)** H194S, **(G)** Q136F, **(H)** A17S and **(I)** Q136S. Data represent mean ± SD of n = 3 technical replicates.
